# A Case of Three Upper Extremity Lesions of Mobile Encapsulated Fat Necrosis in One Patient

**DOI:** 10.7759/cureus.78080

**Published:** 2025-01-27

**Authors:** Connor Sheehan, Theresa Tran, Mehri Mollaee, Sylvia Hsu

**Affiliations:** 1 Dermatology, Temple University Hospital, Philadelphia, USA; 2 Clinical Pathology and Laboratory Medicine, Temple University Hospital, Philadelphia, USA

**Keywords:** benign skin condition, mobile encapsulated fat necrosis, nodular cystic fat necrosis, skin nodule, subcutaneous nodule

## Abstract

Mobile encapsulated fat necrosis is a benign condition that presents as a freely mobile nodule or cluster of nodules beneath the skin. We report a case of an elderly female with a complex medical history who presented with three mobile lesions on the left upper extremity shortly after undergoing a leg amputation, all determined to be mobile encapsulated fat necrosis at different stages of formation. This unusual presentation has not been well described in the literature and highlights the potential for associations with specific underlying medical conditions.

## Introduction

Mobile encapsulated fat necrosis or nodular-cystic fat necrosis refers to small, solitary, asymptomatic subcutaneous lesions that commonly appear on the extremities as a result of local trauma [1]. Due to their small size, these lesions often go unnoticed for many years, and therefore the direct instance of the antecedent injury may be difficult to identify. After an insult occurs, the underlying adipose tissue undergoes necrosis and becomes contained within a cystic structure. With movement and manipulation over time, the blood supply becomes interrupted, and the nodules may detach beneath the skin to become freely mobile [1,2]. Treatment is provided at the request of the patient and is performed via simple surgical excision [3].

## Case presentation

A 67-year-old woman with a complex medical history including peripheral vascular disease, type 2 diabetes mellitus, rheumatoid arthritis managed with oral prednisone, systemic lupus erythematosus (SLE) treated with hydroxychloroquine, asthma treated with mepolizumab, frequent falls, atrial fibrillation, and pulmonary embolism presented with a two-month history of three discrete bumps on her left dorsal hand and left dorsal forearm. She noted that these lesions had first appeared shortly after undergoing an amputation of her right lower extremity in August due to a gangrenous infection. Of note, she had been using a wheelchair since her amputation. On exam, there were three, asymptomatic, freely mobile subcutaneous nodules on the left dorsal hand (Video 1), left proximal dorsal forearm (Video 2), and left distal dorsal forearm (Video 3). All lesions were freely movable by palpation. There was no overlying erythema, induration, or exudate. The patient requested the lesions be removed, for which three incisional biopsies were performed. A clinical diagnosis of mobile encapsulated fat necrosis was made, based on the physical exam.

**Video 1:** Video demonstrating the manipulation of the lesion on the dorsal left hand

**Video 2:** Video demonstrating the manipulation of the lesion on the left proximal dorsal forearm

**Video 3:** Video demonstrating the manipulation of the lesion on the left distal dorsal forearm

**Figure 1:**
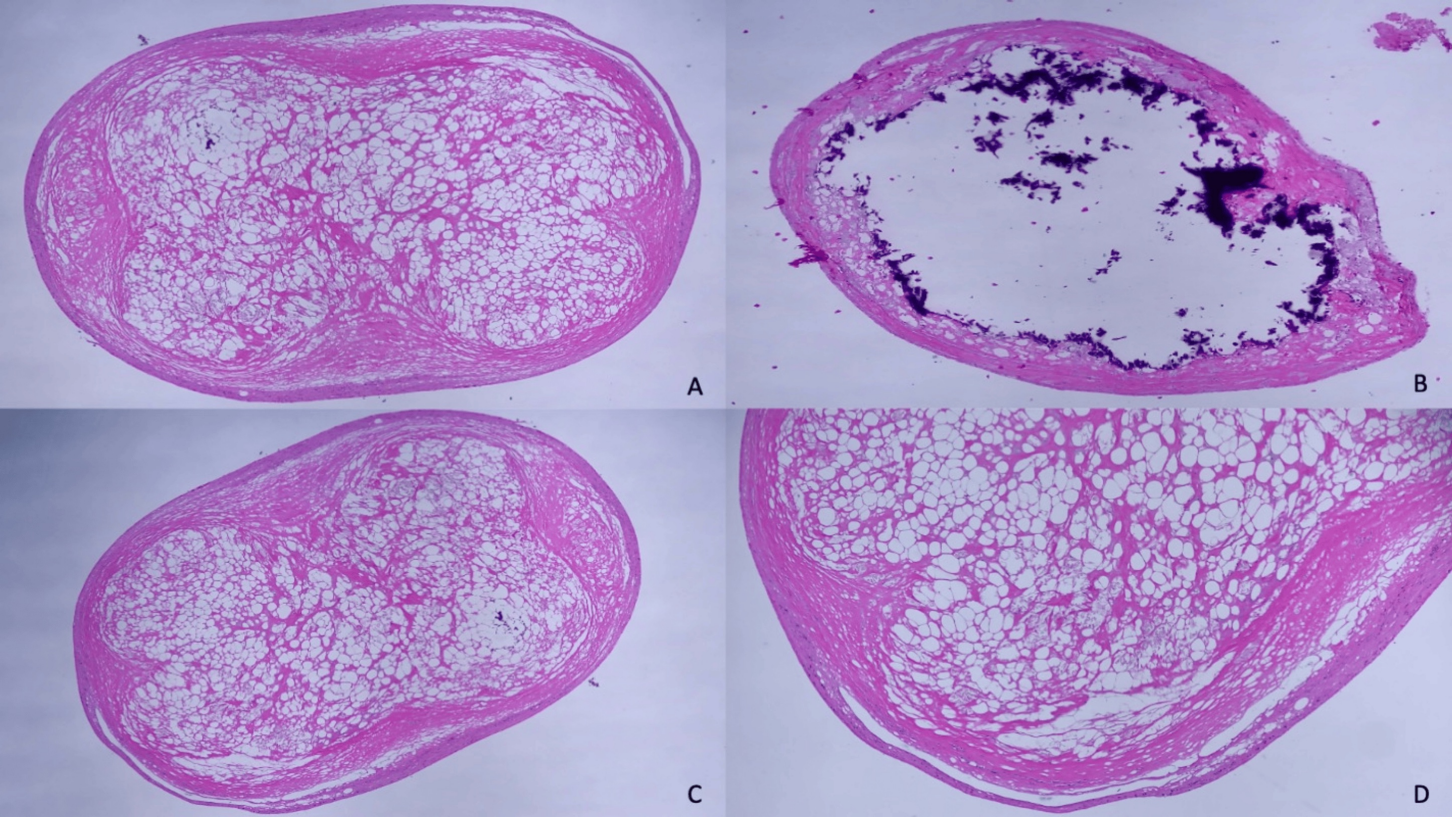
Histopathologic sections of mobile encapsulated fat necrosis Lesion 1 H&E at 20x (A). Lesion 2 H&E at 20x (B). Lesion 3 H&E at 20x (C). Lesion 3 H&E at 40x (D)

Histopathology confirmed the diagnosis for all three sites, showing lesions composed of lobules of necrotic fat surrounded by a thin fibrous hyalinized capsule along with lipomembranous changes as characterized by lipophages and focal inflammatory cells seen near the site (Figure 1). Several weeks later, the patient developed cellulitis of the left upper extremity secondary to the biopsies and was successfully treated with antibiotics. Additional nodules were absent on examination.

## Discussion

This patient had an extensive history of medical conditions and multiple risk factors for repeated trauma due to prior amputations, frequent falls, and continual use of a wheelchair. Additionally, several vascular comorbidities, such as peripheral artery disease and acute thromboembolic events, suggest that her baseline healing capacity may have been impaired. 

Mobile encapsulated fat necrosis most commonly appears in areas of the skin susceptible to trauma, especially the lower extremities [1]. Often, these lesions are grouped together or exist as daughter cysts within a larger area and are seldom found on the upper extremities. It is important to distinguish this benign sequestration of injured adipose tissue from more concerning diagnoses when presented with a similar case. The differential diagnosis for similar subcutaneous lesions includes lipoma, angiolipoma, follicular cyst, ganglion cyst, foreign body granuloma, panniculitides, membranous fat necrosis, and pancreatic fat necrosis [3-5]. The extent of free mobility seen in this patient’s nodules is a reassuring clinical sign that this lesion does not represent an alternative underlying neoplastic process.

Histopathology is a key factor in distinguishing mobile encapsulated fat necrosis from other differential diagnoses, showing an avascular fibrous capsule encasing various stages of fat necrosis and calcification [1]. Some lesions may appear with capillary blood supply within the capsules, while others are completely walled off [3], suggesting a clinical course that involves interruption of blood flow and disconnection from underlying tissue. This patient may have suffered an expedited course of cyst formation and interrupted blood supply secondary to more recent trauma due to her medical history.

## Conclusions

We discussed an unusual presentation of mobile encapsulated fat necrosis in an elderly patient manifesting as three lesions appearing simultaneously in the upper extremity. Further research is needed to investigate the factors that influence the chronicity of lesion formation and associations with underlying hypercoagulable states such as SLE-associated antiphospholipid syndrome, although this was not explicitly ruled out in this patient. It is important that clinicians in other specialties can recognize the features of this skin finding and triage appropriately.
